# Editorial: Promoting nervous system regeneration by treatments targeting neuron-glia interactions

**DOI:** 10.3389/fncel.2023.1355469

**Published:** 2024-01-11

**Authors:** Silmara De Lima, Bruno Siqueira Mietto, Vinicius Toledo Ribas, Victor Tulio Ribeiro-Resende, Alexandre Leite Rodrigues Oliveira, Kevin K. Park

**Affiliations:** ^1^Boston Children's Hospital, Harvard Medical School, Boston, MA, United States; ^2^F.M. Kirby Neurobiology Center, Boston Children's Hospital, Harvard Medical School, Boston, MA, United States; ^3^Department of Molecular and Cellular Biology, Faculty of Arts and Sciences, Harvard University, Cambridge, MA, United States; ^4^Department of Neurology, Boston Children's Hospital, Harvard Medical School, Boston, MA, United States; ^5^Department of Biology, Institute of Biomedical Science, Juiz de Fora Federal University, Juiz de Fora, Minas Gerais, Brazil; ^6^Morphology Department, Institute of Biomedical Science, Federal University of Minas Gerais, Belo Horizonte, Minas Gerais, Brazil; ^7^Biophysics Department, Federal University of Rio de Janeiro, Rio de Janeiro, Brazil; ^8^Biology Institute, State University of Campinas, Campinas, São Paulo, Brazil; ^9^Department of Ophthalmology and Neuroscience, University of Texas Southwestern Medical Center, Dallas, TX, United States

**Keywords:** PNS, CNS, axon regeneration, glial cells, neuronal survival, neuron-glia crosstalk

Puzzled by the differences on how the central and peripheral nervous system behave when challenged, researchers are pursuing the intriguing long standing unanswered question: “can we heal the injured nervous system back to its original function?” As it seems to happen, the nervous system presents major obstacles and tissue-related characteristics, with regards to its regenerative capacity. While some repair can spontaneously occur after peripheral nervous system (PNS) injury, the regenerative capacity of the central nervous system (CNS) is limited. Interestingly, studies on PNS regeneration suggest that Schwann cells coordinate the healing process and adopt a cellular phenotype that favors removal of debris, neuronal survival, axon regeneration, remyelination, transfer of cargos, among many other aspects (Mietto et al., 2015, 2021; Jessen and Mirsky, 2019; Babetto et al., 2020; Bombeiro et al., 2020a,b; Sardella-Silva et al., 2021). Conversely, perturbed Schwann cells metabolism is linked to axonal pathology (Viader et al., 2013; Girardi et al., 2023). Therefore, regeneration of PNS has taught us many lessons, some of them reviewed in *Neuron-Schwann interaction in peripheral nervous system homeostasis, disease, and preclinical treatment* (Oliveira et al.). PNS regeneration has also inspired investigators to study the similarities and differences between the CNS and PNS after a lesion or in the course of neurodegenerative diseases. For more insights, see: *Glia from central and peripheral nervous system are differentially affected by paclitaxel chemotherapy and neurodegenerative properties* (Klein et al.). Others focus on understanding how glial cells within the CNS respond to the stimulation arising from signaling pathways known to stimulate neurogenesis, reported in: *Activation of cannabinoid type 1 receptor modulates oligodendroglial process branching complexity in rat hippocampal cultures stimulated by olfactory ensheathing glial-conditioned medium* (Paes-Colli et al.). On the other hand, very little is known about neuron-glia interaction which affects CNS regeneration. Therefore, investigations into the regenerative process in the CNS needs a broader approach, to show how specific therapies tested in preclinical studies interfere with the regenerative microenvironment (neuronal and glial cells)—as it is shown in *Neuroprotection by upregulation of the major compatibility complex class I in SOD*^*G*93*A*^ *mice* (Tomiyama et al.), where the authors describe that the upregulation of the major histocompatibility complex of class I (MHC I) after interferon beta treatment, at different concentrations, affects spinal motoneuron survival, astrocytic response, microglial activation, synapse modulation, and motor function, in an ALS disease model. In the past decade, great efforts were made to prove that the intrinsic growth capacity of mature CNS neurons could be stimulated and that they could regenerate and reconnect with specific targets after an injury. These efforts led many labs to contribute with evidences that, this is achievable, to some extent, with treatments that start either in the acute or chronic phase after the injury (Kurimoto et al., 2010; Sun et al., 2011; de Lima et al., 2012; Lim et al., 2016; Yungher et al., 2017; Xie et al., 2022). Unfortunately, however, there is a lack of data on the role that glial cells play in this process and, also, whether their interaction can be beneficial or detrimental to the process. Following those studies, it has been shown that regenerating axons can become myelinated (de Lima et al., 2012; Lu et al., 2012; Marin et al., 2016). However, depending on the treatment there is no spontaneous myelination of regenerating axons, but myelination can be stimulated after using a pro-myelination treatment (Wang et al., 2020). There is also evidence that astrocytic scar formation at the injury site supports axon regeneration (Anderson et al., 2016) and stimulation of the growth intrinsic capacity of adult neurons in the retina induces formation of newly formed astrocytes in the regenerating optic nerve (Ribeiro et al., 2022), and that complement cascade at the injury site is required for axon regeneration (Peterson et al., 2021). These are some important evidences that glial cells are active in the process of CNS recovery. These cells play a major role in neuronal integrity and homeostasis, and undoubtedly can cause and/or contribute to axonal pathology during disease conditions. The comprehension of this intricate neuron-glia interaction may provide the basis for promising therapies to repair the nervous system and boost its regenrative capacity after an injury, or prevent neurodegenerative conditions associated with dysfunction of glial cells.

## Author contributions

SD: Conceptualization, Project administration, Supervision, Writing – original draft, Writing – review & editing. BM: Writing – original draft, Writing – review & editing. VR: Writing – review & editing. VR-R: Writing – review & editing. AO: Writing – review & editing. KP: Writing – review & editing.
